# Age of Menarche and Knowledge about Menstrual Hygiene Management among Adolescent School Girls in Amhara Province, Ethiopia: Implication to Health Care Workers & School Teachers

**DOI:** 10.1371/journal.pone.0108644

**Published:** 2014-09-30

**Authors:** Teklemariam Gultie, Desta Hailu, Yinager Workineh

**Affiliations:** 1 Department of Midwifery, Arba Minch University, Arba Minch, Ethiopia; 2 Department of Nursing, Arba Minch University, Arba Minch, Ethiopia; Karolinska Institutet, Sweden

## Abstract

**Background:**

Effective menstrual hygiene has direct and indirect effect on achieving millennium development goals two (universal education), three (gender equality and women empowerment) and, five (improving maternal health). However, in Ethiopiait is an issue which is insufficiently acknowledged in the reproductive health sector. The objective of this study therefore, is to assess the age of menarche and knowledge of adolescents about menstrual hygiene management in Amhara province.

**Method:**

School based cross sectional study was conducted from November 2012 to June 2013. Multistage stage sampling technique was used. The school was first clustered in to grades & sections and thenparticipants were selected by lottery method. A pretested &structured questionnaire was used. Data were entered, cleaned and analyzed using SPSS version 16.0. Finally, multivariate analysis was used to assess independent effect of predictors.

**Findings:**

In this study, 492 students were included, making a response rate of 100%. Mean age at menarche was 14.1±1.4 years. The main sources of information about menstrual hygiene management were teachers for 212 (43.1%). Four hundred forty six (90.7%) respondents had high level knowledge about menstrual hygiene management. Most of the respondents 457 (92.9%) and 475 (96.5%) had access for water and toilet facility respectively. Place of residence (AOR = 1.8, 95%CI: [1.42–1.52]) and educational status of their mothers’ (AOR = 95%CI: [1.15–13.95]) were independent predictors of knowledge about menstrual hygiene management.

**Conclusion:**

Knowledge of respondents about menstrual hygiene management was very high. School teachers were the primary source of information. Place of residence and their mother’s educational status were independent predictors of menstrual hygiene management. Thus, the government of Ethiopia in collaboration with its stalk holders should develop and disseminatereproductive health programmes on menstrual hygiene management targeting both parents and their adolescents. Moreover, parents should be made aware about the need to support their children with appropriate sanitary materials.

## Introduction

### Background

The term adolescence is defined as a period of life between 10–19 years [1], [2]. It is a unique period of age characterized by significant physical, cognitive, emotional and social changes [3]. It is a time when development sets the course for either a healthy or unhealthy adulthood [4]. Every year, about 1.4 million adolescents die worldwide and most of these deaths occur in low & middle income countries. Globally, about 2.2 million adolescents are living with HIV [5]. In Ethiopia, 12% adolescents are already mothers for their first child. About 39% of young women had sex before age 18 and HIV prevalence among 15–24 years old adolescents was 0.3% [2]. Traditionally, these group of people are commonly regarded healthy [6]. However, it is a period when health problems that have serious immediate and long term consequences could first emerge [7], [8] as rapid changes occur before adequate experience of life is achieved [8], [9]. It is a time of vulnerability and thus, health care information obtained from different sources is collectively instrumental in enabling adolescents make healthy decisions [10].

### Problem statement

In females, this period of adolescence is marked with onset of menstruation [11]. In adolescents who experienced menstruation for the first time, menstrual hygiene management (MHM) is constrained by practical, social, economic and cultural factors such as the expense of commercial sanitary pads, lack of water and latrine facilities, lack of private rooms for changing sanitary pads, and limited education about the facts of menstrual hygiene [11]. Adolescents enter puberty unprepared and the information they receive is often selective and surrounded by taboos. In many curricula, there is emphasis on the reproductive process but not on the practical issues [4].

During menstruation, adolescent girls are faced with challenges related to the management of menstrual hygiene in public places. UNICEF estimates that 1 in 10 school age African girls do not attend school during menstruation. Similarly, World Bank statistics indicated that students have been absent from school 4 days every 4 weeks because of menstruation [4].

### Rationale of the study

Addressing menstrual hygiene management directly contributes to Millennium Development Goal-5 (MDG) on improving maternal health. Due to its indirect effect on school absenteeism and gender discrepancy, poor menstrual hygiene and management may seriously hamper the realization of MDG-2 on universal education and MDG-3 on gender equality and women empowerment [12]. However, MHM is an issue that is insufficiently acknowledged in Ethiopian.

Lack of information, misconceptions and adverse attitudes to menstruation may lead to a negative self-image among girls who are experiencing menses for the first time and the culture of silence around menstrual hygienefurther increases the perception of menstruation as something shameful that needs to be hidden [4]. Thus, to break the silence of a taboo and successfully manage menstrual hygiene, adolescents need to understand thebiologic change they are experiencing and be equipped with the skills to cope with it [4]. However, in Ethiopia studies addressing knowledge about menstrual hygiene are scarce. Thus, the objective of the study is to assess the knowledge level and contextual factors related to menstrual hygiene management among young women. Knowledge of the characteristics of this population can be used to develop and evaluate programs and policies.

## Methods and Materials

### Study setting and period

This study was conducted from November 2012 to June 2013 in Mehalmeda secondary school, Mehalmeda town, Amhara province. The town is located 180 Km from Addis Ababa, the capital city of Ethiopia. It is located at an altitude of 3,070 meters above sea level. In the town, there are one secondary school and one district hospital. According to the 2007 Ethiopian census report, the district had a total population of 120,469, of which 29,640 were reproductive age women. The town had a total population of 11,055 and 5,719 of them were females [13]. In 2012/2013 academic year, the school had a total of 3,110 students ranging from grade 9–12. Among those students, female adolescents accounted 1,665.

### Study design and population

A school based cross-sectional study involving quantitative method was undertaken among randomly selected adolescent female students who were attending secondary and preparatory schools during the time of data collection. The study populations of this study were female students in grades 9, 10, 11 & 12 in Mehalmeda high school during the 2012/13 academic year. Young people with learning disability andstudents attending evening classes were excluded. In Ethiopian context, students attending evening classes have different social characteristics from those who regularly attend. Thus, they were excluded from the study to avoid over or under estimation of the study findings.

### Sample size determination and sampling procedure

Single population proportion formula was used to calculate the required sample size. Proportion of knowledge about menstrual hygiene management, margin of error, confidence interval, design effect and non-response rate were assumed to be 50%, 5%, 95%, 1.5 and 5%, respectively.

Taking the above assumption, the sample size became 384. However, the study population was less than 10,000 and a population correction formula was used. Finally, adding 5% none response rate, the sample size was determined to be 492.

Since no previous study was done on knowledge about menstrual hygiene management among comparable society, 50% proportion was considered.

Multistage sampling technique was used to select the study subjects. First, stratification was made in to grades 9, 10, 11, and 12. Then, grades were further stratified by section. Calculated sample size was proportionally allocated to each grade and section according to their number of students. Then, frames of students were developed from student roster of each grade in collaboration with instructors of respective classes. Eligible students were selected using simple random sampling technique from the existing samplingframe (students’ roster). In every step of selection, simple random sampling technique was used.

### Data collection instrument and procedures

Structured self-administered questionnaire was developed for data collection on the variables needed. The questionnaire was developed after thorough literature review was conducted (14, 15, 16) and finally, it was adapted to the local context. The English version questionnaire was translated to Amharic and to check its consistency it was again translatedback to English by experts of both languages. The questionnaire included variables on socio-demographic characteristics, age at menarche, menstrual hygiene materials and knowledge about menstrual hygiene management such asthe use of sanitary materials, ideal adsorbents, frequency at which sanitary pads should be changed, health and social effect of poor menstrual hygiene. Before the actual data collection, the questionnaire was pre-tested on 10% of the study subjects in the neighboring Zemero district high school on a total of 50 students. Based on the findings of the pretest, the tool was modified and finally, the Amharic version questionnaire was administered.

### Data quality management

Data quality was ensured during collection, coding, entry and analysis. Before the actual data collection, pretest was done to check the validity of the instrument. During data collection, one BSc Nurse coordinator and three Diploma Nurse data collectors with previous experience were recruited and adequate training and follow up was provided. Codes were given to the questionnaires during data collection so that errors could betimely addressed. The filled questionnaires were checked for completeness and consistency by the data collectors, supervisors and principal investigator on a daily basis. The data were further cleaned by visualizing and calculating frequencies using SPSS version16 statistical software. Corrections were made according to the original data.

### Data processing and analysis

Data were checked manually for completeness, then coded and entered into Epi Info version 3.5.1, and exported to SPSS version16 for analysis. Descriptive analyses were executed for each of the variables. Bivariate analysis was performedto see the crude association of the independent variables with the outcome variable. Finally, variables which showed significant association with the dependent variable on the bivariate analysis were entered to multivariate logistic regression model to identify their independent effects. P-value and 95% confidence interval (CI) for odds ratio (OR) were used in judging the significance of the associations. P-value less than 0.05 was taken as significant association.

### Measurement

The instrument used to assess knowledge about menstrual hygiene management was designed by all authors in consultation with senior research experts. Most items were developed from a review of literature on knowledge about menstrual hygiene management. Other items were added after formal conversation was made with senior researchers and reproductive health experts. Then, some of the items for which the researchers labeled having low validity wereexcluded and the final instrument containing 8 items was developed. In addition, internal consistency (reliability) among the items was computed andCronbach’s alpha was determined to be 0.84. Finally, respondents who scored 50% and above were considered as having high level knowledge; whereas, those with a score of less than 50% wereconsidered as having low level knowledge about menstrual hygiene management.

### Ethical consideration

Ethical clearance was obtained from Addis Ababa University, department of Nursing and Midwifery institutional review board (IRB). The letter of collaboration was written from department of Nursing and Midwifery to Mehalmeda secondary school for the main study and to Zemero secondary school for the pretest. The school director was briefed on the objectives of the study. Each study participant was adequately informed about the purpose, benefits and risks of the study and their right to discontinue or refuse to participate in the study. Finally, written informed consent was secured from each study participants and their confidentiality, privacy and anonymity was maintained. For respondents whose age less than 18 years old, written informed assent was obtained from their parents/guardians.

## Result

### Socio-demographic characteristics of participants

Four hundred ninety two female students were participated in the study, making a response rate of 100%. The mean age of the study participants and mean age of menarche were 16.85±1.336 and 14.73±1.451 years respectively. Among the participants, 369 (75%) were in the age group of 16–18 years and 315 (64%) were grade 9 followed by 123 (25%) from grade 10. Among the respondents, 483 (98.2%) were orthodox christian and 490 (99.6%) were from Amhara ethnic group. The majority of the respondents 476 (96.7%) were single and 285 (57.9%) were urban dwellers. Three hundred twenty seven (66.5%) girls were living with their father and mothers. The parents of 349 (70.9%) participants were farmers and 241 (49%) of the respondents’ parents earn less than 500 Ethiopian birr per month (table 1).

**Table 1:** Socio-demographic characteristic of female students in Mehalmeda secondary school, Amhara region, Ethiopia, June 2013.

Variables		Frequency	Percentage
Age at interview	13–15 years	85	17.3
	16–18 years	369	75.0
	19–21 years	36	7.3
	>21 years	2	0.4
Age at menarche	9–12 years	44	8.9
	13–16 years	411	83.5
	>17 years	37	7.5
Education status of respondents	grade 9	315	64.0
	grade 10	123	25.0
	grade 11	40	8.1
	grade 12	14	2.8
Place of residence	Urban	285	57.9
	Rural	207	42.1
Religion	Orthodox	483	98.2
	Protestant	7	1.4
	Muslim	2	0.4
Ethnicity	Amhara	490	99.6
	Others	2	0.4
Marital status	Single	476	96.7
	Married	16	3.3
Respondents living arrangement	Mother and father	327	66.5
	Mother only	60	12.2
	Father only	14	2.8
	Relatives	25	5.1
	Friends	16	3.3
	Alone	50	10.2
Fathers’ educational status	Can’t read and write	71	14.4
	Read and write	300	61.0
	Elementary school	53	10.8
	High school	33	6.7
	College	35	7.1
Mothers’ educational status	Can’t read and write	142	28.9
	Read and write	257	52.2
	Elementary school	56	11.4
	High school	20	4.1
	College	17	3.5
Mothers’ occupation	House wife	19	3.9
	Farmer	349	70.9
	Trade/business	71	14.4
	Employed (Government/private)	53	10.8
Family monthly income	<500 birr	241	49.0
	500–1000 birr	133	27.0
	1000–1500 birr	62	12.6
	>1500 birr	56	11.4

### Source of information about menstrual hygiene

The main source of information about menstrual hygiene was teachers for 212 (43.1%) followed by mother 113 (22.96%). One hundred eighty nine (38.4%) adolescents reported that they had ever discussed with their friends about menstrual hygiene. One hundred twenty seven (25.8%) didn’t learn about menstrual hygiene in their class and 167 (33.9%) reported they never discussed about menstrual hygiene with any one (table 2).

**Table 2:** Source of information about menstrual hygiene among female students in Mehalmeda secondary school, Amhara region, Ethiopia, June 2013.

Variables		Frequency	Percentage
Learned in class about menstrual hygiene	Yes	365	74.2
	No	127	25.8
Ever discussed about menstrual hygiene	Yes	325	66.1
	No	167	33.9
Respondents discussed about menstrual hygiene with*	Friend	189	38.4
	Mother	43	8.7
	Sister	70	14.2
	Teacher	38	7.7
Source of information about menstrual hygiene*	Teacher	212	43.1
	Mother	113	22.96
	Friend	73	14.8
	Media	58	11.58

*Some respondents have multiple responses.

### Knowledge of respondents about menstrual hygiene management

The main menstrual hygiene management materials known by most 242 (49.18%) respondents were commercially made sanitary pads followed by 186 (37.8%) underwear and 88 (17.88%) homemade pad. Three hundred fifty two (71.5%) participantsreported that there is foul smellingduring menstruation and 337 (68.5%) said menstrual blood is unhygienic. Four hundred nineteen (85.2%) participants reported that poor menstrual hygiene predisposes toan infection. Four hundred forty six (90.7%) and 431 (87.6%) participants responded that use of sanitary pad and genital wash respectively should be done frequently. Generally, four hundred forty six (90.7%) respondents had high level knowledge; whereas, 46 (9.3%) scored low level knowledge about menstrual hygiene management (table 3).

**Table 3:** Knowledge of menstrual hygiene among female students in Mehalmeda secondary school, Amhara region, Ethiopia, June 2013.

Variables		Frequency	Percentage
Sanitary material that can possibly be used duringmenstruation*	Clean homemade pad orcloth	88	17.88
	Commercially madesanitary pad	242	49.18
	Underwear	186	37.8
Absorbent w/c is ideally used duringmenstruation	Commercially madesanitary pad	429	87.2
	Home made with cloth piece	63	12.8
Uncared menstruation produces foul odor	No	140	28.5
	Yes	352	71.5
Menstruation in early adolescence is not normal	No	155	31.5
	Yes	337	68.5
Poor menstrual hygiene predispose to infection	No	73	14.8
	Yes	419	85.2
Pad should be changed frequently	No	46	9.3
	Yes	446	90.7
External genitalia should be washed with water& soap frequently	No	61	12.4
	Yes	431	87.6
Personal hygiene prevents menstrual pain	No	116	23.6
	Yes	376	76.4
Knowledge of respondents about menstrualhygiene	High level knowledge	446	90.7
	Low level knowledge	46	9.3

*Some respondents have multiple responses.

### Sociocultural and physical environment for menstrual hygiene management

Most of the respondents 457 (92.9%) and 475 (96.5%) hadaccess for water and toilet facility respectively. The privacy of the school toilet was kept for 407 (82.7%) respondents (table 4).

**Table 4:** Sociocultural and environmental factors for the management of menstrual hygiene among female students in Mehalmeda secondary school, Amhara region, Ethiopia, June 2013.

Variables		Frequency	Percentage
Had access to clean water	No	35	7.1
	Yes	457	92.9
Had access to toilet	No	17	3.5
	Yes	475	96.5
Privacy of the toilet is kept	No	71	14.4
	Yes	407	82.7
Feel comfortable in schoolwhile menstruating	No	341	69.3
	Yes	151	30.7
Reason for being uncomfortablein school*	No place to dispose used pad	49	10.0
	No private place to changesanitary pad	193	39.2
	No water for washing	94	19.1
	I had Pain or discomfort	11	2.2
Absent from school	No	240	48.8
	Yes	252	51.2
Menstruation interferes schoolperformance	No	291	59.1
	Yes	201	40.9
Activities restricted duringmenstruation	No	175	35.6
	Yes	317	64.4

*Some respondents have multiple responses.

### Place of disposal for used menstrual hygiene materials

**Figure 1:**
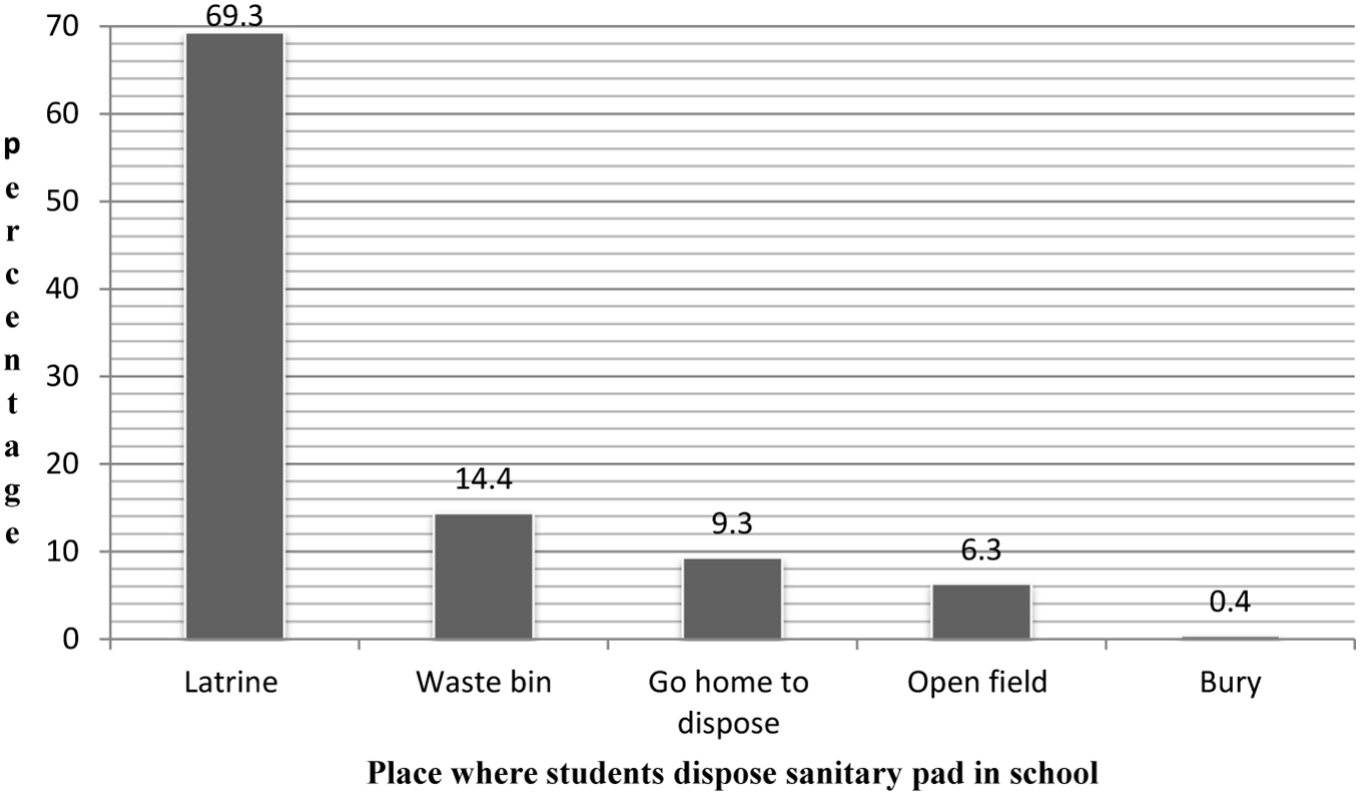
Place of disposal for menstrual hygiene materials among students in Mehalmeda secondary school, Amhara region, Ethiopia, June 2013.

Three hundred forty one (69.3%) and 31 (6.3%) participants reported that they disposed used pad in school latrine and open field respectively. Majority of the respondents 341 (69.3%) feltuncomfortable being in school during menstruation due to lack of private place to change sanitary pad by193 (39.2%) and absence of water for washing by 94 (19.1%). About half of the ladies 252 (51.2%) were absent from school during menstruation and among those, 125 (25.4%) were absent only one day per cycle (Figure 1).

### School absenteeism status of students during menstruation

**Figure 2:**
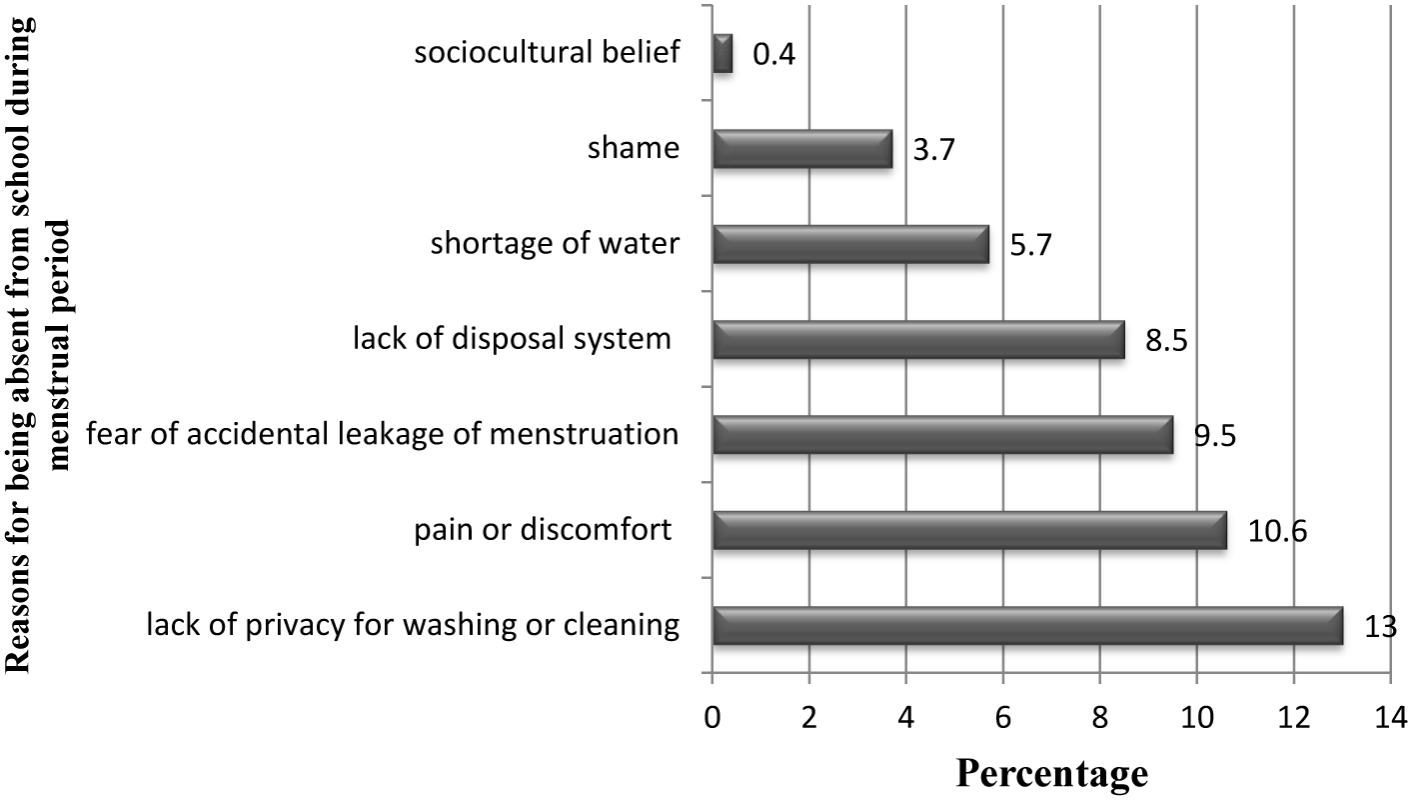
Reason for being absent from school during menstruation among female students in Mehalmeda secondary school, Amhara region, Ethiopia, June 2013.

The main reason for school absenteeism during menstruation was lack of privacy for washing or cleaning mentioned by 64 (13%), pain or discomfort by 52 (10.6%) and fear of accidental leakage of menstrual blood by 47 (9.5%). Poor menstrual hygiene interfered students school performance as reported by 201 (40.9%) respondents. Among those who were restricted, religious activities were mentioned by 193 (39.2%) study participants (Figure 2).

### Factors affecting knowledge of respondents about menstrual hygiene management

In multivariable logistic regression analysis, educational status of respondents’ mothers and place of residence were found to be independent predictors of female students’ knowledge about menstrual hygiene management (Table 5).

**Table 5:** Factors affecting level of knowledge about menstrual hygiene management among female students in Mehalmeda secondary school, Amhara region, Ethiopia, June 2013.

Variables		Knowledge about MHM		Crude OR	Adjusted OR
		High	Low		
Age at interview	<18 years	408(82.9%)	46(9.3%)	1	1
	>18 years	38(7.7%)	0(0.00%)	0.00(0.00–5.10)	0.00(0.00–7.62)
Place of residence	Urban	262(53.3%)	23(4.7%)	0.7(0.38–1.29)	**1.80(1.42–1.52)** 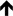
	Rural	184(37.4%)	23(4.7%)	1	**1**
Monthly income	500	216(43.9%)	25(5.1%)	1	1
	500–1000	122(24.8%)	11(2.2%)	0.78(0.37–1.64)	0.88(0.41–1.90)
	>1000	108(22%)	10(2%)	0.80(1.37–1.74)	1.24(0.54–2.86)
Education level ofrespondents	Grade 9	282(57.3%)	33(6.7%)	1	1
	Grade 10	111(22.6%)	12(2.4%)	0.92(0.46–1.85)	0.94(0.46–1.92)
	Grade 11	39(7.9%)	1(0.20)	0.22(0.03–1.65)	0.21(0.03–1.67)
	Grade 12	14(2.8%)	46(9.3)	0.002(0.00–1.30)	0.00(0.00–1.57)
Respondents livingarrangement	Parents	229(60.8%)	28(5.7%)	0.58(0.24–1.40)	0.52(0.20–1.32)
	Motheronly	55(11.2%)	5(1.0%)	0.56(0.17–1.88)	0.62(0.17–2.21)
	Father/relatives/friends	49(10%)	6(1.2%)	0.75(0.24–2.41)	0.85(0.25–2.90)
	Alone	43(8.7%)	7(1.4%)	1	1
Mother’s educationalstatus	No formal education	360(73.2%)	39(7.9%)	1	1
	Elementary	49(10%)	7(1.4%)	1.32(0.56–3.10)	**4(1.15–13.95)** 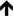
	Secondary & above	37(7.5%)	0.0(0.0%)	0.001(0.0–8.20)	0.00(0.0–1.38)
Fathers’ educationalstatus	No formal education	330(67.1%)	41(8.3%)	1	1
	Elementary	50(10.2%)	3(0.6%)	0.48(0.14–1.62)	0.25(0.05–1.18)
	Secondary & above	66(13.4%)	2(0.4%)	0.24(0.06–1.03)	0.17(0.03–1.03)
Ever discussed aboutMHM	No	155(31.5%)	12(2.4%)	1	1
	Yes	446(90.7%)	46(9.3%)	1.51(0.76–3.00)	1.85(0.90–3.79)

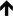
Statistically significant at p<0.05.

In this study, place of residence was found to have statistical association with level of knowledge about menstrual hygiene management. Urban residents were about 2 times more likely to be knowledgeable about menstrual hygiene management thanthose adolescents living in the rural setting (AOR = 1.8, 95%CI: [1.42–1.52]). The other strong predictor was educational status of respondents’ mothers. The likelihood of having high knowledge about menstrual hygiene management was higher in respondents whose mothers’ educational status was elementary than those whose mothers did not attend formal education (AOR = 95%CI: [1.15–13.95]).

## Discussion

This facility based cross-sectional study tried to assess the level of knowledge of female high school students about menstrual hygiene management and identified associated factors in Mehalmeda high school, Amhara province, Ethiopia.

The present study revealed that the mean age of menarche was found to be 14.7±1.4 years. This is congruent with findings from south Eastern Zone of Tigray, Ethiopia [17]. However, it is higher than the study findings from Ghana, Egypt, Saudi Arabiya, India, Japan [14], [18], [19], [20], [21], [22], [23]. This difference could be attributed to the influence of both heredity and socioeconomic conditions, mainly nutrition. Girls from upper socio-economic class have reached menarche at an earlier age than those from the lower socio-economic class as they attain a certain critical weight at an early age due to improved nutrition and better health.

In line with evidence from Mumbai [24], this study indicated that majority of the female respondents had good knowledge about menstrual hygiene management. In contrast to report from study conducted in Nepal, Nagpur, India, Nigeria [15], [16], [22], [25], this study demonstrated that knowledge of menstrual hygiene among female high school students was high. This might be attributed to the time gap that accessibility, availability and ability to optimally utilize reproductive health information may be improved as time increases.

Generally, it is desirable to have a school teacher or a health worker to be the first source of information ensuring that right knowledge and skill has been imparted to the adolescents. Evidence also showed that the quality of educators can have a huge impact on sexuality education including information being provided related to menstruation [4]. In contrast to evidences from India, Nagpur, Tigray, Nigeria where mothers of respondents were major source of information [8], [16], [17], [24], this study showed that the primary sources of information about menstrual hygiene management were school teachers. Thus, it is important to ensure that school teachers who deliver information and education on sexuality receive adequate training and ongoing support and supervision. Evidence showed that when teachers receive training and support in their role as sexuality educators they feel more comfortable and equipped to provide effective, objective and nonjudgmental sexuality education [4]. In addition, it should be ensured that a variety of context specific teaching methods are used in providing sexuality education to young people.

In agreement with study findings from Nagpur and India [16], [26], this study indicated that respondents who resided in urban are more likely to have good knowledge of menstrual hygiene management than their counter parts of rural residents. This difference could be explained by the fact that the proportion of urban residents could have accessible reproductive health care service, and better decision making autonomy than rural female students. In addition, this inconsistency might also be attributable to the difference in implementation of relevant health intervention programs.

Similarly, this study revealed that educational status of female students’ mothers was found to be independent predictor of knowledge about menstrual hygiene. Female students whose mother had attended elementary school had higher odds of having good knowledge about menstrual hygiene management as compared to students whose mother had never attended formal education. This is comparable with findings from Mumbai and Nigeria [24], [27]. This can partly be explained by the fact that educated women have better access to health service information, improved perceptions towards menstrual hygiene and can utilize health careservice information optimally.

Lack of latrine and water supply seriously affects menstrual hygiene management and jeopardizes physical and psychological health of school adolescents. However, in this study water and toilets facility of the school were almost fully accessible to the students. Thus, this implies that if adolescents have good knowledge and positive attitude towards their own menstrual hygiene management they have an opportunity to easily practice it and the risk of developing poor menstrual hygiene related reproductive health problems is very low.

## Conclusion

Based on the findings of the present study, it was concluded that the knowledge of female adolescentstudents about menstrual hygiene management was very high. Almost all female students have full access to clean water and latrine facility. School teachers followed by mothers of respondents were the primary sources of information. From among the risk factors, place of residence and their mother’s educational status were found to be independent predictors of menstrual hygiene management. Thus, urban residence and having a formally educated mother are opportunities in which school girls can learn, avoid taboos and develop sufficient knowledge.

### Recommendations

Based on the findings of the present study, it was recommended that:

- Knowledge influences attitude and practice over time. The government and development partners should therefore work towards further developing and disseminating sensitive programme targeted at both parents and the adolescents on the unmet needs of adolescents including sexuality education. The electronic and print media, community organizations and faith-based organizations are authentic means of disseminating these messages. Moreover, there is also a need for programmes and interventions targeting parents to explore parents child communication.
- Parents should be made to acknowledge the need to support their children at school with sanitary menstrual materials in addition to other basic hygienic products. The government should on the other compliment these provisions as part of the school health programs.
- Finally, additional studies should be done using a wider geographic scope and a larger sample size including their mothers in order to produce sufficient and comprehensive information.
